# 552 DFB: Tiny burn, big problem - the implications of a diabetic foot burn

**DOI:** 10.1093/jbcr/irac012.180

**Published:** 2022-03-23

**Authors:** Stanley Uche, Rita Gayed, Rohit Mittal, Walter L Ingram

**Affiliations:** Burrell college of osteopathic medicine, Las cruces, New Mexico; Grady Health System, Atlanta, Georgia; Emory University, Atlanta, Georgia; Emory / Grady Burn Unit, Atlanta, Georgia

## Abstract

**Introduction:**

In the United States, >30 million people (10.5% of the population) have diabetes, both diagnosed and undiagnosed. Many of these patients go on to develop diabetes related complications, such as peripheral neuropathy. Patients with diabetes are also prone to foot injury. The purpose of this study is to determine clinical outcomes associated with foot burns in patients with diabetes.

**Methods:**

A retrospective chart review of adult patients (≥18yo) admitted to a major metropolitan burn center at a safety-net hospital from 2008-2021 with an isolated burn to the lower extremity and a diagnosis of diabetes mellitus. Patients were categorized based on admission hemoglobin A1C. The primary outcome was hospital length of stay and secondary outcomes were time to presentation, infection, amputations, and mortality.

**Results:**

A total of 136 patients were included in the study, 79% of which were male.84% of the patients were < 65yo and the mean age was 54.1yo and an average HbA1C of 9%. Scald injury was most common mechanism of injury (54%) followed by radiant (24.3%) and contact burns (16.2%). The average burn size was 2.3% TBSA. The median length of stay was 7 days (3 days per percent TBSA). Patients presented on average 5.2 days following injury with 44.8% patients presenting with an infection. More than half (54%) of the patients had peripheral neuropathy at baseline. A majority (74%) of the patients underwent surgical excision. About 18% of the patients underwent an amputation and 3.7% were admitted to the intensive care unit with an average ICU length of stay of 7 Additionally, there was 1 inpatient mortality.

**Conclusions:**

Our study found that lower extremity burns in patients with diabetes were associated with a prolonged hospital stay, high infection rate, need for surgical intervention and high morbidity/disability rate as evident by the number of patients requiring amputations despite the small size of the burn. Peripheral neuropathy may be one of the reasons leading to delayed presentation to a burn center following injury. Hence, burn prevention in this patient population through intense education on proper foot care and inspection along with adequate glycemic control are key to improving outcomes for patients with diabetes.

